# Chemical Composition of *Eriodictyon californicum* (California Yerba Santa) Cultivated in Ontario, Oregon, USA

**DOI:** 10.3390/molecules31081356

**Published:** 2026-04-21

**Authors:** Clinton C. Shock, Ambika Poudel, Prabodh Satyal, Jianping Zhao, Joseph Lee, Mei Wang, William N. Setzer

**Affiliations:** 1 Department of Crop and Soil Science, Oregon State University, Ontario, OR 97914, USA; clinton.shock@gmail.com; 2 Aromatic Plant Research Center, 230 N 1200 E, Suite 100, Lehi, UT 84043, USApsatyal@aromaticplant.org (P.S.); 3 National Center for Natural Products Research, School of Pharmacy, University of Mississippi, University, MS 38677, USAjclee1@olemiss.edu (J.L.); 4 Natural Products Utilization Research Unit, Agricultural Research Service, United States Department of Agriculture, University, MS 38677, USA; mei.wang@usda.gov; 5 Department of Chemistry, University of Alabama in Huntsville, Huntsville, AL 35899, USA

**Keywords:** essential oil, monoterpenoids, polyphenolics, flavonoids, eriolic acid C, sterubin

## Abstract

Leaves from California yerba santa (*Eriodictyon californicum*) have been used historically by indigenous peoples for medicinal purposes. Recent research has ascribed potential pharmaceutical effects to leaf polyphenols, without a consideration of other constituents. Based on prior analyses of polyphenols in leaves sampled in nature, five accessions known to be rich in sterubin and five accessions known to be rich in eriolic acid C were grown from seeds in Ontario, Oregon, and samples of their leaves were harvested and evaluated for their essential oil and polyphenol contents. The major essential oil components in *E. californicum* were 1,8-cineole (0.6–35.5%), (*Z*)-β-ocimene (6.8–15.7%), terpinen-4-ol (8.3–16.1%), α-pinene (2.6–13.6%), β-phellandrene (1.9–11.7%), γ-terpinene (4.6–7.9%), ethyl (*E*)-cinnamate (0.2–8.9%), α-terpineol (1.5–5.2%), *p*-cymene (2.0–5.3%), and β-pinene (0.6–6.8%). Fifteen polyphenols with a prominence of eriolic acid C, rosmarinic acid, sterubin, homoeriodictyol, 6-methoxynaringenin, hesperetin, and eriodictyol were identified. Essential oils may contribute to the medicinal properties of the leaves of California yerba santa. Results from the ten samples were evaluated for both polyphenols and essential oils; the variations in several essential oils may be correlated to variations in some of the polyphenols.

## 1. Introduction

*Eriodictyon californicum* (Hook. & Arn.) Torr. (syn. *Eriodictyon californicum* (Hook. & Arn.) Decne.), Namaceae, California yerba santa, is native to California and southwestern Oregon where it grows in chaparral habitats of the coastal and interior mountains [1,2,3]. Several California Native American tribes used the plant to treat colds, upper respiratory infections (whooping cough, pneumonia, tuberculosis), asthma, rheumatism, and externally to treat sores [4].

Several biologically active phytochemical constituents have been isolated from leaf extracts of *E. californicum* and identified, including the polyphenolics rosmarinic acid, salvianolic acid H, melitric acid A, and eriolic acids A-D and the flavonoids chrysoeriol, cirsimaritin, eriodictyol, naringenin, 6-methoxynaringenin, hispidulin, homoeriodictyol, jaceosidin, hesperetin, sterubin, sakuranetin, 6-methoxysakuranetin, and pinocembrin [5,6,7,8,9,10]. Interestingly, homoeriodictyol, sterubin, and eriodictyol decrease the bitter taste of compounds such as caffeine or quinine [11], while sakuranetin, 6-methoxysakurenetin, and jaceosidin are antagonists of the human bitterness receptor [10]. The distribution, botany, phytochemistry, traditional and current folk uses, and biological activities of *E. californicum* have been reviewed [12].

The purpose of this research project is to (a) demonstrate the potential for cultivation of *E. californicum* in a more extreme high desert environment, (b) determine whether the polyphenolic/flavonoid profiles of cultivated *E. californicum* are comparable to wild populations, and (c) to obtain the essential oils of *E. californicum*. As far as we are aware, the essential oil of *E. californicum* has not been investigated. In this work, the essential oil compositions as well as the polyphenolic components from 10 accessions of *E. californicum* cultivated in Ontario, Oregon, are presented.

## 2. Results

The hydrodistillation of the *E. californicum* samples produced pale yellow essential oils in yields ranging from 0.80% to 3.40%. A gas chromatographic analysis allowed for the identification of 165 components in the essential oils, accounting for 98.8–99.6% of the total compositions. Although there were remarkable variations in the essential oil compositions, the essential oils were dominated by monoterpene hydrocarbons and oxygenated monoterpenoids, particularly menthane and acyclic monoterpenoids. The major components in the *E. californicum* essential oils (Table 1) were 1,8-cineole (0.6–35.5%), (*Z*)-β-ocimene (6.8–15.7%), terpinen-4-ol (8.3–16.1%), α-pinene (2.6–13.6%), β-phellandrene (1.9–11.7%), γ-terpinene (4.6–7.9%), ethyl (*E*)-cinnamate (0.2–8.9%), α-terpineol (1.5–5.2%), *p*-cymene (2.0–5.3%), and β-pinene (0.6–6.8%). The complete essential oil compositions are compiled in Supplementary Table S1. Chiral GC-MS was carried out to discern the enantiomeric distributions of chiral monoterpenoid components in *E. californicum* essential oils (Table 2).

A total of 15 polyphenolics (four phenolic acids and eleven flavonoids) were quantified in the *E. californicum* samples (Table 3). The compounds with the highest concentrations in the samples were rosmarinic acid (21.99–52.94 mg/g), eriolic acid C (0.67–151.96 mg/g), homoeriodictyol (25.92–61.47 mg/g), and sterubin (0.00–71.80 mg/g). Although the concentrations of rosmarinic acid and homoeriodictyol were relatively consistent within the 10 samples, both eriolic acid C and sterubin showed a wide variation.

## 3. Discussion

### 3.1. Eriodictyon californicum Chemotypes

**Figure 1:**
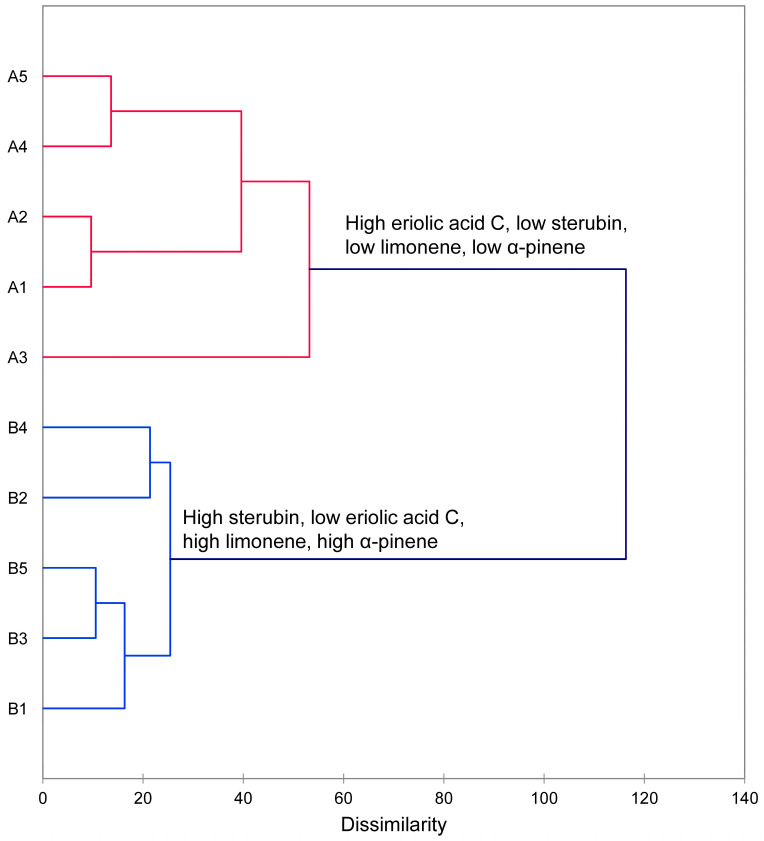
The dendrogram obtained from the agglomerative hierarchical cluster analysis (HCA) of *Eriodictyon californicum* based on the normalized concentrations of the major essential oil components and the polyphenolic components.

**Figure 2:**
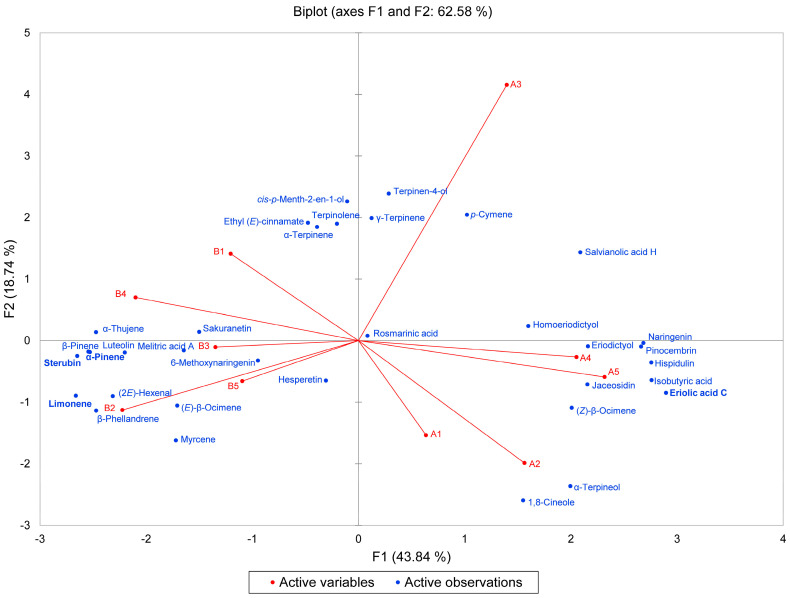
The bidimensional plot of the first two components (F1 and F2) from the principal component analysis (PCA) of *Eriodictyon californicum* samples, based on the normalized concentrations of the major essential oil components and the polyphenolic components. “A” points are the high-eriolic-acid-C plants; “B” points are the high-sterubin plants.

In the essential oil compositions, several components showed a wide variation between the samples (e.g., α-pinene, β-phellandrene, and 1,8-cineole), which suggests that there may be different chemotypes of *E. californicum* based on essential oil profiles. Furthermore, Wang and co-workers have described two chemotypes of *E. californicum* based on concentrations of polyphenolics [9]. As noted in Wang et al., there were wide concentration variations in several polyphenolic components, particularly sterubin, which is also noted in the present study. Based on the very limited number of samples in these potential chemotype evaluations, multivariate analyses (HCA and PCA) were carried out (Figure 1 and Figure 2). The HCA shows two clearly defined clusters: (1) a cluster with high eriolic acid C, low sterubin, low limonene, and low α-pinene levels and (2) a cluster with high sterubin, low eriolic acid C, high limonene, and high α-pinene levels. The PCA corroborates the HCA and shows the association of one group with eriolic acid C and the other group associated with sterubin, limonene, and α-pinene.

### 3.2. Correlation Between Essential Oil Composition and Polyphenolic Composition

A Pearson correlation was carried out to examine potential correlations between essential oil components and polyphenolic components (Supplementary Table S2). 1,8-Cineole does not show significant correlations with any of the polyphenolic compounds. However, α-pinene shows a significant negative correlation with eriolic acid C (−0.854, *p* = 0.002). β-Phellandrene (−0.817, *p* = 0.004) and β-pinene (−0.833, *p* = 0.003) also show significant negative correlations with eriolic acid C. Conversely, α-pinene (0.746, *p* = 0.013), β-phellandrene (0.732, *p* = 0.016), and β-pinene (0.747, *p* = 0.013) show significant positive correlations with sterubin. On the other hand, (*Z*)-β-ocimene (0.784, *p* = 0.007) and α-terpineol (0.691, *p* = 0.027) show positive correlations with eriolic acid C, but (*Z*)-β-ocimene is negatively correlated with sterubin (−0.805, *p* = 0.005).

It is not clear why there should be a correlation between polyphenolic concentrations and monoterpenoid concentrations. The monoterpenoids are biosynthesized by either the mevalonic acid (MVA) pathway or the 2-methyl-d-erythritol-4-phosphate (MEP) pathway [18,19], while flavonoids, rosmarinic acid, salvianolic acid H, and melitric acid A are biosynthesized via the phenylpropanoid pathway [20,21]. The biosynthesis of eriolic acid C has not been reported in the literature. However, based on its structure, eriolic acid C may arise via a polyketide-like assembly followed by downstream prenyl/terpenoid coupling (see, for example, [22]). However, correlated differences in monoterpenoids and flavonoids may be due to transcription factors or transcription factor modules that coordinately regulate genes in both biosynthetic pathways. For example, in grape (*Vitis vinifera*) berries, flavonol (quercetin and kaempferol glycosides) and monoterpenoid (α-terpineol, citronellol, geraniol, and nerol) levels increased with an increasing *MYB24* gene expression [23]. In addition, flux adjustments in terpene biosynthesis can influence flavonoid precursors and vice versa. For example, the knockdown of the limonene synthase gene expression in spearmint (*Mentha spicata*) resulted in a reduction in limonene and carvone biosynthesis but an increase in flavonoid and polyphenolic metabolites [24].

### 3.3. Enantiomeric Distribution of Monoterpenoids

Enantioselective GC-MS indicated that the major enantiomers were (−)-α-thujene (92.1 ± 0.7%), (−)-sabinene (88.9 ± 2.5%), (−)-α-phellandrene (58.1 ± 7.0%), (−)-limonene (57.6 ± 13.7%), (+)-β-phellandrene (88.7 ± 4.0%), (−)-*cis*-sabinene hydrate (88.7 ± 2.7%), (+)-linalool (72.8 ± 6.5%), (−)-*trans*-sabinene hydrate (89.8 ± 1.6%), and (−)-terpinen-4-ol (74.5 ± 2.1%). Furthermore, these enantiomeric distributions were consistent between the two different chemotypes (eriolic acid and sterubin chemotypes). In contrast, however, α-pinene showed a different enantiomeric distribution in the two chemotypes: 51.0 ± 6.0% (−)-α-pinene in the high-eriolic-acid chemotype but only 18.0 ± 9.0% in the high-sterubin chemotype. Analogous differences were noted for (+)-camphene (54.5 ± 3.7% and 68.1 ± 9.2% for the two chemotypes, respectively), (−)-β-pinene (72.3 ± 3.0% and 33.4 ± 6.9%, respectively), and (−)-α-terpineol (81.0 ± 6.5% and 55.1 ± 15.0%, respectively). Differential gene regulation in the two different chemotypes likely accounts for the differences in enantiomeric distributions for α-pinene, camphene, β-pinene, and α-terpineol.

The enantiomeric distributions can be affected by the geographical location and environment. For example, sabinene, limonene, and α-terpineol distributions were notably different for *Ericameria nauseosa* (rubber rabbitbrush) samples collected from Idaho and Utah [25]. Likewise, *Pinus albicaulis* (whitebark pine) essential oils from California and Wyoming showed different distributions of limonene and β-phellandrene [26]. Variations in linalool and terpinen-4-ol enantiomeric distributions have been noted for the thymol/carvacrol chemotype and the linalool/α-terpineol/bornyl acetate chemotype of *Origanum vulgare* [27]. Two chemotypes of key lime (*Citrus aurantium*) showed slightly different distributions of α-phellandrene, β-phellandrene, linalool, and α-terpineol [28].

### 3.4. Biological Activities of Eriodictyon californicum Components

*Eriodictyon californicum* has been traditionally employed in Native American medicine for inflammatory and respiratory ailments [4]. The Atsugewi people of northern California chewed the plant to treat coughs and colds and used the leaves and branches to prepare a steam bath to treat rheumatism. Likewise, the Miwok Native Americans took an infusion of the leaves and flowers to treat coughs and colds; a poultice of the leaves was used on aching or sore spots.

The major polyphenolic components in *E. californicum* identified in this study were eriolic acid C, homoeriodictyol, rosmarinic acid, sterubin, hesperetin, 6-methoxynaringenin, and eriodictyol. Of these, the flavonoids eriodictyol, hesperetin, homoeriodictyol, and sterubin are well known for various biological activities. For example, eriodictyol has demonstrated potent antioxidant, anti-inflammatory [29], neuroprotective [30], cardioprotective, hepatoprotective, and anticancer properties [31]. Likewise, hesperetin has shown antioxidant and anti-inflammatory [32], neuroprotective [33], cardioprotective [34], hepatoprotective [35], anticancer [36], and antimicrobial effects [37]. Homoeriodictyol has shown antinociceptive potential in animal models [38]. Sterubin has exhibited robust antioxidant, anti-inflammatory, and neuroprotective properties, which may prove useful for the prevention and treatment of Parkinson’s disease and Alzheimer’s disease [39,40,41]. The polyphenolic compound rosmarinic acid has been investigated for its antioxidant and anti-inflammatory effects [42,43], antimicrobial activity [44], and neuroprotective effects [45].

Complementing the biological activities of the polyphenolic components, some of the major essential oil components of *E. californicum* have also shown biological activities of medicinal importance. 1,8-Cineole, terpinen-4-ol, and α- and β-pinene have shown antibacterial activity against respiratory-tract-infection-causing pathogens [46,47]. Furthermore, 1,8-cineole has shown mucolytic activity [48] and respiratory system protection properties by inhibiting inflammatory factors and inflammatory cells [49,50,51]. Both 1,8-cineole and β-pinene have shown antinociceptive properties [52]. Terpinen-4-ol has shown antibacterial and antibiofilm activities against *Staphylococcus aureus* [53].

## 4. Materials and Methods

### 4.1. Chemicals

Reference standards for flavonoids (n = 11) and phenolic compounds (n = 4) were obtained for quantitative analysis. Eriodictyol, naringenin, sakuranetin, hesperetin, luteolin, and homoeriodictyol were purchased from ChromaDex Inc. (Irvine, CA, USA). Both pinocembrin and rosmarinic acid were obtained from Indofine (Township, NJ, USA). Sterubin was from BOC Science (Shirley, NY, USA). Jaceosidin was purchased from Cayman Chemicals (Ann Arbor, MI, USA). 6-Methoxynaringenin was obtained from ChemFaces (Wuhan, China). Hispidulin was purchased from PhytoLab (Vestenbergsgreuth, Germany). Melitric acid A, salvianolic acid H, and eriolic acid C were isolated at The National Center for Natural Products Research (NCNPR), University of Mississippi. The purity of the eriolic acid C was >95%, while the purities of melitric acid A and salvianolic acid H were 92%. Both the purity and identity of these compounds were confirmed by chromatographic data as well as HRMS and NMR spectral analysis.

### 4.2. Plant Materials

Based on prior polyphenol analyses of leaves sampled in nature, seeds from five distinct accessions known to be rich in eriolic acid C (A1–A5) and seeds from five distinct accessions known to be rich in sterubin (B1–B5) were obtained from chapparal habitats in western California; the precise locations of these plants are not revealed in order to protect the critical populations from overexploitation. The plants were grown in Ontario, Oregon. Seeds were treated to stimulate germination, and the resulting plants were transplanted in early June 2024 to a silt loam field in Ontario, Oregon. Plants were hand weeded and irrigated with drip irrigation. The plant tops (up to 20 cm foliage, post-flowering) were harvested from cultivated plants on 24 and 28 October 2024. The fresh plant samples were frozen (−20 °C) and stored frozen until processed.

### 4.3. Essential Oils

The fresh/frozen plant materials were chopped and hydrodistilled using a Likens–Nickerson apparatus, with continuous extraction of the distillate with dichloromethane, for 6 h to produce the essential oils (Table 4). The mass of the essential oil was obtained using an analytical balance after evaporation of the dichloromethane using a stream of dry air.

### 4.4. Gas Chromatographic Analysis

The *E. californicum* essential oils were analyzed via gas chromatography–mass spectrometry (GC-MS) and enantioselective GC-MS as previously described [54,55]. The detailed instrumentation and protocols are summarized in Supplementary Table S3. Retention indices were determined using the method of van den Dool and Kratz [13]. Essential oil components were determined by comparing mass spectral fragmentation patterns and retention index values with those reported in the databases of Adams [14], Satyal [15], Mondello [16], and NIST20 [17]. Component percentages were calculated based on manual peak integration without correction factors.

### 4.5. Polyphenolic Analysis

The polyphenolic acids and flavonoids in the foliage of *E. californicum* were extracted with methanol, identified by UHPLC/ESI/QtoF, and quantified by UHPLC/DAD as previously described [9].

#### 4.5.1. UHPLC/DAD/Q-ToF Analysis

An Agilent (Agilent Technologies, Santa Clara, CA, USA) 1290 Infinity Series UHPLC system was used for sample analysis. The instrument was equipped with a binary pump, autosampler, thermostatted column compartment, and a diode array detector (DAD). Separation was achieved using a mobile phase consisting of water (A) and acetonitrile (B), which both contained 0.1% formic acid. The following gradient elution at a flow rate of 0.2 mL/min was used: 0 min 20% B, 0–12 min 31% B, 12–24 min 36% B, 24–29 min 72% B, 29–32 min 100% B. After each sample, a 5 min wash of 100% B was completed followed by an equilibration period of 6 min with 5% B. An Agilent Poroshell EC-C_18_ (2.1 × 150 mm, 2.7 µm) column maintained at 30 °C was used to achieve separation. The DAD was programmed to collect wavelengths at 220, 260, 288, 330, and 340 nm.

Mass spectrometric analysis was performed with an Agilent G6545B Q-ToF MS/MS using an ESI source. The following parameters were used throughout the experiment: fragmentor voltage 100 V, capillary voltage 3500 V, nebulizer pressure 35 psi, sheath gas flow rate of 11 L/min at a temperature of 350 °C, and drying gas (N_2_) flow rate of 10 L/min at a temperature of 320 °C. Samples were analyzed over the mass range of *m*/*z* 100–1100 in negative mode. Instrument parameters and data acquisition were controlled by Agilent MassHunter Acquisition Software (v. A.05.01). MassHunter Qualitative and Quantitative Analysis software (both v. B.10.00) were used for data analysis.

#### 4.5.2. Calibration Curve Preparation for Quantification

Stock solutions of each standard were prepared at a concentration of 5 mg/mL in methanol (hesperidin was dissolved in methanol with the aid of 10 drops of DMSO). The stock solutions were diluted in order to obtain calibration concentrations ranging from 0.5 to 500 µg/mL (500, 300, 200, 100, 75, 50, 25, 10, 7.5, 5, 1, and 0.5 µg/mL). Duplicate injections of each calibration standard were used to calculate the calibration curve slope, intercept, and *R*^2^ values. Supplementary Figure S1 and Table S4 present the UV spectra, extracted ion chromatograms (EICs) in ESI^−^ mode, and ToF MS spectra for each compound under optimized conditions. The deprotonated molecular ions [M−H]^−^ were consistently observed as the base peaks and served as the basis for quantification.

### 4.6. Statistical Analysis

Multivariate analyses (hierarchical cluster analysis, HCA, and principal component analysis, PCA) were carried out using the percentages of the 19 most abundant essential oil components (average > 1.0%) and the concentrations (mg/g) of the 15 polyphenolic compounds. The data were normalized by subtracting the mean of each component and dividing by the standard deviation. The HCA and PCA were carried out using XLSTAT v. 2018.1.1.62926 (Addinsoft, Paris, France). For the HCA, the normalized component values were used. Dissimilarity was used to determine clusters based on Euclidean distance, and Ward’s method was used to define agglomeration. For the PCA, the Pearson correlation type was used using the same normalized data. The Pearson correlation coefficients (r) were calculated to determine the relationship between the essential oil components and the polyphenolic components.

## 5. Conclusions

This research has shown that *Eriodictyon californicum* (California yerba santa) contains bioactive polyphenolics and essential oil components consistent with its traditional use in treating respiratory and inflammatory ailments, which play a role in the current use of the plant as a nutraceutical. Two distinct chemotypes of *E. californicum* were identified in this study based on the concentrations of eriolic acid C and sterubin in addition to essential oil components such as α-pinene, β-pinene, and limonene. One chemotype showed high eriolic acid C levels (140.9 ± 11.8 mg/g) but low sterubin (0.22 ± 0.47 mg/g), low α-pinene (3.1 ± 0.5%), low β-pinene (0.8 ± 0.2%), and low limonene (1.1 ± 0.4%) concentrations, and the other chemotype had high sterubin (58.05 ± 13.46 mg/g), low eriolic acid C (3.95 ± 3.61 mg/g), high α-pinene (10.4 ± 3.5%), high β-pinene (5.1 ± 2.2%), and relatively high limonene (3.6 ± 0.8%) levels. A major finding in this study is that polyphenol chemotypes in nature appeared to retain their chemotypes when cultivated from seeds at a different location. That is, low- and high-sterubin plants gave rise to low- and high-sterubin plants when cultivated in new locations, which was the same for eriolic acid C. However, a broader range of samples should be evaluated for chemotype variations. The different chemotypes likely influence the plant’s biological activities and, therefore, its suitability for herbal medicinal use. This study provides a basis for the traditional use of *E. californicum* and highlights its potential for further development as a nutraceutical. However, in vivo, clinical, and toxicological studies are needed to evaluate the therapeutic utility of this herbal medicine. Additionally, further research is needed in order to understand the biosynthesis and gene regulation of its biologically active components.

## Figures and Tables

**Table 1:** Key chemical components (percentages, averages > 1.0%) in the foliar essential oils of *Eriodictyon californicum* (California yerba santa).

RI_calc_	RI_db_	Compounds	A1	A2	A3	A4	A5	B1	B2	B3	B4	B5
766	774	Isobutyric acid	1.3	2.0	1.8	3.8	2.1	0.3	0.1	0.1	0.1	0.6
850	850	(2*E*)-Hexenal	0.7	0.8	0.9	1.2	0.3	0.8	2.9	1.4	2.5	2.2
926	927	α-Thujene	1.0	0.8	1.1	1.1	0.5	1.4	1.4	1.9	1.9	1.4
933	933	α-Pinene	3.1	2.6	3.6	3.6	2.8	12.9	13.6	5.6	11.9	7.7
977	978	β-Pinene	0.9	0.6	1.0	0.9	0.6	6.7	6.8	2.2	6.5	3.1
989	991	Myrcene	1.1	1.2	0.8	1.7	0.9	1.2	2.0	1.4	1.6	1.4
1017	1018	α-Terpinene	2.6	2.3	3.8	2.8	2.0	2.8	2.4	3.1	3.4	2.2
1025	1025	*p*-Cymene	2.0	2.2	5.3	3.6	3.8	4.3	2.2	2.2	3.0	3.1
1030	1030	Limonene	1.1	1.0	0.7	1.8	1.0	2.8	4.8	3.7	3.7	2.8
1031	1031	β-Phellandrene	3.2	3.0	2.6	1.9	4.4	4.4	11.7	8.6	7.9	7.7
1033	1031	1,8-Cineole	35.5	34.2	0.8	16.3	25.1	6.8	3.0	22.7	0.6	16.2
1036	1034	(*Z*)-β-Ocimene	13.5	15.7	12.9	13.4	10.9	8.8	10.7	6.8	10.9	9.9
1046	1045	(*E*)-β-Ocimene	2.1	2.6	2.1	2.4	1.9	2.4	4.0	2.0	3.3	2.2
1058	1058	γ-Terpinene	5.3	4.7	7.9	5.7	4.6	5.3	4.9	5.9	6.4	4.6
1085	1087	Terpinolene	1.0	0.8	1.4	1.1	0.8	1.0	0.9	1.1	1.3	0.8
1125	1124	*cis-p*-Menth-2-en-1-ol	0.8	0.8	2.3	1.1	1.3	1.4	1.3	1.3	1.4	1.2
1181	1180	Terpinen-4-ol	8.5	8.3	16.1	10.4	10.7	12.1	8.9	11.1	12.0	8.6
1195	1195	α-Terpineol	5.2	5.0	1.6	3.7	4.9	2.3	1.7	3.7	1.5	2.3
1466	1469	Ethyl (*E*)-cinnamate	0.2	0.2	8.5	3.0	0.8	8.9	2.6	1.5	2.5	5.0

RI_calc_ = Retention index determined with respect to a homologous series of *n*-alkanes using the arithmetic formula of van den Dool and Kratz [13]. RI_db_ = Reference retention index values from the databases [14,15,16,17].

**Table 2:** Enantiomeric distribution (percentages) of chiral monoterpenoids in the essential oils of *Eriodictyon californicum* (California yerba santa).

Enantiomers	RI_calc_	RI_db_	A1	A2	A3	A4	A5	B1	B2	B3	B4	B5
(+)-α-Thujene	951	950	7.0	8.7	8.1	8.7	8.6	6.4	7.9	7.4	7.8	7.8
(−)-α-Thujene	952	951	93.0	91.3	91.9	91.3	91.4	93.6	92.1	92.6	92.2	92.2
(−)-α-Pinene	975	976	53.1	59.9	48.7	43.7	49.7	12.3	10.5	32.1	13.5	21.8
(+)-α-Pinene	980	982	46.9	40.1	51.3	56.3	50.3	87.7	89.5	67.9	86.5	78.2
(−)-Camphene	1000	998	42.8	47.1	44.5	42.0	51.2	23.3	26.7	45.2	26.6	37.8
(+)-Camphene	1004	1005	57.2	52.9	55.5	58.0	48.8	76.7	73.3	54.8	73.4	62.2
(+)-Sabinene	1021	1021	11.0	9.4	5.9	12.0	14.8	10.3	14.1	11.4	10.2	12.3
(−)-Sabinene	1030	1030	89.0	90.6	94.1	88.0	85.2	89.7	85.9	88.6	89.8	87.7
(+)-β-Pinene	1026	1027	28.5	24.6	30.3	30.6	24.4	74.6	68.1	57.6	70.9	61.5
(−)-β-Pinene	1032	1031	71.5	75.4	69.7	69.4	75.6	25.4	31.9	42.4	29.1	38.5
(−)-α-Phellandrene	1051	1050	63.4	61.0	61.5	64.1	62.7	60.8	41.2	53.9	53.7	59.1
(+)-α-Phellandrene	1052	1053	36.6	39.0	38.5	35.9	37.3	39.2	58.8	46.1	46.3	40.9
(−)-Limonene	1075	1073	68.0	68.2	71.4	47.5	77.4	36.0	50.8	46.6	46.7	63.3
(+)-Limonene	1081	1081	32.0	31.8	28.6	52.5	22.6	64.0	49.2	53.4	53.3	36.4
(−)-β-Phellandrene	1084	1083	13.0	12.3	18.2	13.7	12.1	14.0	4.0	7.7	8.5	9.1
(+)-β-Phellandrene	1087	1089	87.0	87.7	81.8	86.3	87.9	86.0	96.0	92.3	91.5	90.9
(+)-*cis*-Sabinene hydrate	1199	1199	14.6	13.3	6.2	12.0	15.0	10.6	11.6	11.0	8.9	9.8
(−)-*cis*-Sabinene hydrate	1201	1202	85.4	86.7	93.8	88.0	85.0	89.4	88.4	89.0	91.1	90.2
(−)-Linalool	1229	1228	21.1	24.0	18.2	29.8	26.6	40.3	21.0	33.1	28.1	29.3
(+)-Linalool	1232	1231	78.9	76.0	81.8	70.2	73.4	59.7	79.0	66.9	71.9	70.7
(+)-*trans*-Sabinene hydrate	1231	1231	9.7	9.9	7.8	10.8	11.9	8.2	12.7	11.3	8.8	10.9
(−)-*trans*-Sabinene hydrate	1235	1235	90.3	90.1	92.2	89.2	88.1	91.9	87.3	88.7	91.2	89.1
(+)-Terpinen-4-ol	1296	1297	25.0	24.9	20.8	26.8	24.0	25.5	28.5	26.9	25.8	26.8
(−)-Terpinen-4-ol	1299	1300	75.0	75.1	79.2	73.2	76.0	74.5	71.5	73.1	74.2	73.2
(−)-α-Terpineol	1347	1347	86.2	86.6	70.9	78.5	82.7	55.1	40.0	73.8	40.6	66.0
(+)-α-Terpineol	1356	1356	13.8	13.4	29.1	21.5	17.3	44.9	60.0	26.2	59.4	34.0

RI_calc_ = Retention index determined with respect to a homologous series of *n*-alkanes using the arithmetic formula of van den Dool and Kratz [13]. RI_db_ = Reference retention index values from our in-house database prepared using commercially available standards.

**Table 3:** Concentrations of polyphenolic components (mg compound/g dry plant material) in *Eriodictyon californicum* (California yerba santa).

Compounds	A1	A2	A3	A4	A5	B1	B2	B3	B4	B5
Rosmarinic acid	38.09	39.80	35.88	23.96	23.82	52.94	22.07	34.03	21.99	31.85
Salvianolic acid H	5.93	9.90	23.58	11.68	9.08	5.88	2.74	4.81	2.98	3.56
Melitric acid A	0.00	0.00	0.00	1.07	0.00	1.02	0.21	3.27	8.46	2.06
Eriodictyol	11.55	13.25	14.50	18.82	14.86	8.41	7.92	11.73	14.28	9.87
Luteolin	1.62	0.62	0.98	n.d.	n.d.	1.16	1.10	2.48	2.83	1.27
6-Methoxynaringenin	25.56	22.17	20.53	2.43	2.16	13.92	10.71	21.99	21.40	17.38
Naringenin	3.84	6.66	6.91	4.37	6.62	2.54	1.89	2.84	2.46	3.72
Hispidulin	0.14	5.04	4.08	5.49	5.14	0.14	0.08	0.14	0.08	0.12
Homoeriodictyol	33.37	30.97	46.38	61.47	57.64	30.02	25.92	46.93	47.86	31.65
Jaceosidin	0.25	0.56	0.51	3.23	1.30	0.00	0.00	0.02	0.01	0.00
Hesperetin	26.32	20.62	19.82	9.26	13.16	13.63	11.57	23.28	20.41	18.81
Eriolic acid C	146.98	141.54	121.01	151.96	143.12	1.30	0.67	6.88	8.71	2.19
Sterubin	0.01	0.01	1.06	0.00	0.00	55.75	39.77	70.76	71.80	52.18
Sakuranetin	7.32	0.00	4.70	0.22	0.00	5.35	3.11	5.25	3.20	6.51
Pinocembrin	0.66	0.73	1.71	1.86	2.89	0.04	0.03	0.07	0.10	0.48

n.d. = not detected.

**Table 4:** Hydrodistillation details for *Eriodictyon californicum*.

*E. californicum* Sample	Mass Foliage (g)	Mass Essential Oil (g)	% Yield ^a^
A1	133.33	3.32	2.49
A2	97.16	1.95	2.00
A3	79.65	1.16	1.46
A4	57.79	1.47	2.54
A5	67.79	0.54	0.80
B1	119.84	2.39	2.00
B2	105.87	2.34	2.21
B3	109.07	3.04	2.79
B4	111.77	3.80	3.40
B5	124.61	3.30	2.65

^a^ % Yield = (100 × mass essential oil)/mass plant material.

## Data Availability

All data are available within the manuscript and Supplementary Tables.

## Supplementary Materials

The following supporting information can be downloaded at https://www.mdpi.com/article/10.3390/molecules31081356/s1, Table S1. Chemical composition (percentages) of *Eriodictyon californicum* (California yerba santa) essential oils. Table S2. Pearson correlation between major essential oil components and polyphenolic components in *Eriodictyon californicum* (California yerba santa). Table S3. Instrument details for the gas chromatographic analyses of *Eriodictyon californicum* (California yerba santa) essential oils. Table S4. UHPLC/DAD/Q-ToF quantification of targeted compounds in *Eriodictyon californicum* samples. Figure S1. UV spectra, extracted ion chromatograms (EICs) in ESI^−^ mode, and ToF MS spectra for each compound quantified in *Eriodictyon californicum*.

## Abbreviations

The following abbreviations are used in this manuscript:

DAD	Diode array detector
DMSO	Dimethylsulfoxide
ESI	Electrospray ionization
GC-MS	Gas chromatography–mass spectrometry
HCA	Hierarchical cluster analysis
MEP	2-methyl-d-erythritol-4-phosphate
MVA	Mevalonic acid
MYB24	Myeloblastosis 24 gene
NIST	National Institute of Standards and Technology
PCA	Principal component analysis
QToF	Quadrupole time-of-flight
RI	Retention index
UHPLC	Ultra-high-performance liquid chromatography
