# Supplementary material for: Chemical Composition of Eriodictyon californicum (California Yerba Santa) Cultivated in Ontario, Oregon, USA
Source: Molecules. 2026 Apr 21;31(8):1356. doi: 10.3390/molecules31081356 (PMC13118867; doi:10.3390/molecules31081356)
Supplement: Supplementary file 1 [file molecules-31-01356-s001.zip › molecules-4254801-supplementary.pdf]

Chemical Composition of *Eriodictyon californicum* (California Yerba Santa) Cultivated in Ontario, Oregon, USA

Clinton C. Shock [1], Ambika Poudel [2], Prabodh Satyal [2], Jianping Zhao [3], Joseph Lee [3], Mei Wang [4], and William N. Setzer [2,5,*]

1 Department of Crop and Soil Science, Oregon State University, Ontario, OR 97914, USA; clinton.shock@gmail.com
2 Aromatic Plant Research Center, 230 N 1200 E, Suite 100, Lehi, UT 84043, USA; psatyal@aromaticplant.org (P.S.)
3 National Center for Natural Products Research, School of Pharmacy, University of Mississippi, University, MS 38677; jclee1@olemiss.edu (J.L.)
4 Natural Products Utilization Research Unit, Agricultural Research Service, United States Department of Agriculture, University, MS 38677; mei.wang@usda.gov
5 Department of Chemistry, University of Alabama in Huntsville, Huntsville, AL 35899, U.S.A
* Correspondence: wsetzer@chemistry.uah.edu

Supplementary Table S1. Chemical composition (percentages) of *Eriodictyon californicum* (California yerba santa) essential oils.

RI_{calc}	RI_{db}	Compounds	A1	A2	A3	A4	A5	B1	B2	B3	B4	B5
766	774	Isobutyric acid	1.3	2.0	1.8	3.8	2.1	0.3	0.1	0.1	0.1	0.6
769	770	Ethyl isoburyrate	-	-	-	-	-	0.6	0.2	0.2	0.2	0.3
788	780	3-Methyl-2-butenal	0.1	0.1	0.1	0.1	0.1	tr	-	-	-	0.1
797	797	(3*Z*)-Hexenal	-	-	-	-	-	tr	0.1	tr	0.1	0.1
798	801	Hexanal	0.1	0.1	0.1	0.1	tr	0.1	0.2	0.1	0.2	0.2
830	830	Isovaleric acid	tr	tr	0.4	0.4	0.3	0.1	tr	tr	tr	0.1
839	840	2-Methylbutyric acid	0.2	0.1	0.2	0.7	0.3	0.1	tr	tr	tr	0.2
840	842	Ethyl 2-methylbutyrate	-	-	0.2	-	-	0.1	0.1	tr	0.1	0.1
849	850	Ethyl isovalerate	-	-	0.2	-	-	0.2	-	-	-	-
850	850	(2*E*)-Hexenal	0.7	0.8	0.9	1.2	0.3	0.8	2.9	1.4	2.5	2.2
921	921	Hashishene	tr	tr	tr	tr	tr	tr	tr	tr	tr	tr
923	923	Tricyclene	tr	tr	tr	tr	tr	0.1	0.1	tr	0.1	tr
926	927	α-Thujene	1.0	0.8	1.1	1.1	0.5	1.4	1.4	1.9	1.9	1.4
931	932	2-Methyl-5-isopropenylfuran	-	-	-	0.1	tr	-	-	-	-	-
933	933	α-Pinene	3.1	2.6	3.6	3.6	2.8	12.9	13.6	5.6	11.9	7.7
936	935	Ethyl tiglate	-	-	0.1	tr	tr	tr	-	-	-	-
946	944	4-Methylpent-2-enolide	0.3	0.3	0.1	0.7	0.3	0.1	0.2	0.1	0.2	0.5
948	948	α-Fenchene	tr	tr	tr	tr	tr	0.1	tr	tr	0.1	tr
949	950	Camphene	0.1	0.1	0.1	0.1	0.1	0.4	0.4	0.2	0.4	0.3
953	953	Thuja-2,4(10)-diene	-	-	-	-	-	0.1	tr	tr	tr	-

RI_{calc}	RI_{db}	Compounds	A1	A2	A3	A4	A5	B1	B2	B3	B4	B5
961	960	Benzaldehyde	tr	tr	tr	tr	tr	tr	tr	tr	tr	tr
972	972	Sabinene	0.2	0.2	0.2	0.2	0.1	0.7	0.8	1.0	0.8	0.8
977	978	β-Pinene	0.9	0.6	1.0	0.9	0.6	6.7	6.8	2.2	6.5	3.1
984	986	6-Methyl-5-hepten-2-one	0.1	0.1	0.1	0.2	0.2	0.1	0.1	tr	0.1	0.1
989	991	Myrcene	1.1	1.2	0.8	1.7	0.9	1.2	2.0	1.4	1.6	1.4
1005	1004	*p*-Mentha-1(7),8-diene	0.2	0.2	0.1	0.2	0.3	0.2	0.7	0.5	0.5	0.5
1007	1007	α-Phellandrene	0.5	0.5	0.7	0.6	0.7	0.5	0.5	0.4	0.6	0.5
1009	1009	δ-3-Carene	-	-	-	-	tr	tr	tr	tr	tr	tr
1017	1018	α-Terpinene	2.6	2.3	3.8	2.8	2.0	2.8	2.4	3.1	3.4	2.2
1021	1022	*p*-Methylanisole	-	-	-	-	-	0.1	0.2	tr	0.1	0.1
1025	1025	*p*-Cymene	2.0	2.2	5.3	3.6	3.8	4.3	2.2	2.2	3.0	3.1
1030	1030	Limonene	1.1	1.0	0.7	1.8	1.0	2.8	4.8	3.7	3.7	2.8
1031	1031	β-Phellandrene	3.2	3.0	2.6	1.9	4.4	4.4	11.7	8.6	7.9	7.7
1033	1031	1,8-Cineole	35.5	34.2	0.8	16.3	25.1	6.8	3.0	22.7	0.6	16.2
1036	1034	(*Z*)-β-Ocimene	13.5	15.7	12.9	13.4	10.9	8.8	10.7	6.8	10.9	9.9
1043	1043	Phenylacetaldehyde	0.1	0.1	0.1	0.2	0.1	0.1	0.3	0.2	0.3	0.4
1046	1045	(*E*)-β-Ocimene	2.1	2.6	2.1	2.4	1.9	2.4	4.0	2.0	3.3	2.2
1058	1058	γ-Terpinene	5.3	4.7	7.9	5.7	4.6	5.3	4.9	5.9	6.4	4.6
1070	1069	*cis*-Sabinene hydrate	0.5	0.4	0.5	0.4	0.3	0.9	1.0	1.2	0.9	0.6
1071	1072	*p*-Cresol	-	-	-	-	-	tr	tr	tr	tr	0.2
1085	1087	Terpinolene	1.0	0.8	1.4	1.1	0.8	1.0	0.9	1.1	1.3	0.8
1090	1093	*p*-Cymenene	0.1	0.1	0.1	0.2	0.2	0.1	tr	tr	0.1	0.1
1091	1091	Rosefuran	0.3	0.3	0.2	0.3	0.3	-	-	-	-	-
1094	1095	Methyl benzoate	-	-	0.2	0.1	0.1	-	-	-	-	-
1100	1101	Linalool	0.9	0.7	0.5	0.9	0.8	0.5	1.1	0.7	0.8	0.4
1101	1101	*trans*-Sabinene hydrate	0.5	0.4	0.7	0.4	0.3	1.0	1.1	1.4	1.1	0.8
1105	1107	Nonanal	-	0.1	tr	0.1	0.1	-	-	tr	tr	tr
1125	1124	*cis*-*p*-Menth-2-en-1-ol	0.8	0.8	2.3	1.1	1.3	1.4	1.3	1.3	1.4	1.2
1128	1127	*allo*-Ocimene	0.5	0.6	0.5	0.7	0.6	0.4	0.4	0.3	0.5	0.4
1137	1138	Benzeneacetonitrile	0.1	0.2	0.3	0.2	0.2	0.2	0.1	0.1	0.1	0.3
1142	1142	*trans*-*p*-Menth-2-en-1-ol	0.6	0.6	1.1	0.8	0.9	0.9	0.8	0.9	0.9	0.8
1152	1149	1,4-Dihydronaphthalene	-	0.1	-	-	-	-	-	-	-	-
1154	1156	Camphene hydrate	-	-	-	-	-	0.1	0.1	tr	0.1	tr
1163	1164	Pinocarvone	-	-	-	-	-	0.1	tr	tr	0.1	tr

RI$_{calc}$	RI$_{db}$	Compounds	A1	A2	A3	A4	A5	B1	B2	B3	B4	B5
1169	1169	Ethyl benzoate	-	-	0.1	-	-	-	-	-	-	-
1170	1170	δ-Terpineol	0.5	0.5	-	0.4	0.7	0.1	tr	0.2	-	0.2
1172	1173	Octanoic acid	0.1	-	0.5	0.2	0.2	-	-	-	-	-
1181	1180	Terpinen-4-ol	8.5	8.3	16.1	10.4	10.7	12.1	8.9	11.1	12.0	8.6
1187	1186	*p*-Cymen-8-ol	0.1	0.1	0.3	0.2	0.3	0.4	0.2	0.2	0.3	0.2
1191	1190	Methyl salicylate	tr	0.1	0.1	0.2	tr	-	-	-	-	-
1195	1195	α-Terpineol	5.2	5.0	1.6	3.7	4.9	2.3	1.7	3.7	1.5	2.3
1207	1208	Verbenone	-	-	-	-	-	0.1	tr	-	0.1	-
1240	1240	4-Phenyl-2-butanone	0.1	0.1	1.3	1.2	0.6	-	-	-	-	-
1254	1254	Piperitone	-	-	-	-	-	-	-	-	0.1	0.1
1272	1273	Methyl hydrocinnamate	-	-	0.1	-	tr	-	-	-	-	-
1277	1277	Phellandral	0.1	0.1	0.1	0.1	0.2	0.1	0.1	0.1	0.1	0.1
1293	1294	2-Undecanone	-	-	0.1	0.1	0.1	-	-	0.1	-	-
1297	1299	*cis*-Theaspirane	0.2	0.2	0.2	0.3	0.3	0.2	0.2	0.1	0.2	0.3
1303	1300	Carvacrol	-	-	-	-	-	0.1	-	tr	0.1	0.1
1303	1305	6-Hydroxycarvotanacetone	tr	tr	0.1	0.1	tr	0.3	0.2	0.1	0.2	0.3
1327	1338	4-Phenyl-2-butanol	0.2	0.5	0.5	0.5	0.2	-	-	-	-	-
1348	1347	Ethyl hydrocinnamate	-	-	1.2	0.5	0.1	0.5	0.1	0.1	0.2	0.3
1350	1350	Citronellyl acetate	-	-	-	-	-	-	0.1	-	0.2	-
1352	1356	*p*-Acetanisole	tr	tr	0.1	-	0.1	0.1	-	0.1	tr	0.1
1370	1370	α-Ylangene	0.1	0.1	tr	0.1	0.1	-	tr	tr	tr	0.1
1373	1374	Methyl *p*-anisate	-	-	0.1	-	tr	-	-	-	-	-
1374	1375	Ethyl (*Z*)-cinnamate	-	-	0.6	0.3	0.1	0.8	0.3	0.2	0.3	0.6
1376	1375	α-Copaene	0.1	0.1	0.1	0.2	0.2	-	0.1	0.1	0.1	tr
1379	1379	(*E*)-β-Damascenone	0.1	0.1	tr	0.2	0.2	tr	tr	tr	tr	tr
1384	1384	Methyl (*E*)-cinnamate	tr	0.1	0.6	0.4	0.1	0.1	0.1	0.1	0.1	0.1
1407	1406	α-Gurjunene	tr	0.1	-	-	-	-	-	-	-	-
1410	1410	Dodecanal	-	-	tr	0.1	tr	-	-	-	-	-
1418	1419	β-Ylangene	0.1	tr	tr	tr	tr	-	tr	tr	tr	-
1419	1418	(*E*)-β-Caryophyllene	-	-	tr	tr	tr	tr	0.1	tr	0.1	tr
1429	1430	γ-Elemene	-	-	-	-	-	-	0.1	0.1	0.1	0.1
1430	1430	β-Copaene	0.1	tr	tr	tr	tr	-	-	-	-	-
1433	1432	*trans*-α-Bergamotene	0.1	0.1	tr	0.1	0.1	-	-	-	-	-
1438	1438	Aromadendrene	0.1	0.1	tr	0.1	0.2	-	-	-	0.1	-

RI_{calc}	RI_{db}	Compounds	A1	A2	A3	A4	A5	B1	B2	B3	B4	B5
1441	1442	Guaia-6,9-diene	-	-	-	-	-	0.1	0.1	0.2	tr	0.3
1447	1447	Geranyl acetone	0.1	0.1	0.1	0.1	0.1	0.1	0.1	tr	0.1	0.1
1448	1448	Ethyl *p*-anisate	-	-	0.1	-	-	-	-	-	-	0.3
1449	1450	*cis*-Muurola-3,5-diene	-	-	-	-	-	-	0.1	0.1	0.1	-
1453	1452	(*E*)-β-Farnesene	-	-	-	-	-	0.1	0.1	0.1	0.1	0.1
1455	1454	α-Humulene	-	-	-	-	-	-	0.1	tr	tr	tr
1459	1458	*allo*-Aromadendrene	0.1	0.1	-	-	0.1	-	-	-	0.1	-
1466	1469	Ethyl (*E*)-cinnamate	0.2	0.2	8.5	3.0	0.8	8.9	2.6	1.5	2.5	5.0
1472	1472	*trans*-Cadina-1(6),4-diene	tr	tr	tr	tr	0.1	-	-	tr	0.1	-
1478	1481	(*E*)-β-Ionone	-	-	-	-	0.1	-	-	-	-	-
1479	1479	α-Amorphene	-	-	-	-	-	-	tr	0.1	tr	0.1
1488	1489	β-Selinene	-	-	-	-	-	-	0.1	0.1	0.1	0.1
1490	1489	(*Z*,*E*)-α-Farnesene	-	-	-	-	-	0.1	0.1	0.1	0.1	tr
1492	1492	*trans*-Muurola-4(14),5-diene	-	-	0.1	-	0.1	-	0.1	0.1	0.1	-
1495	1495	2-Tridecanone	-	-	0.1	0.1	0.1	-	-	0.1	-	-
1496	1497	α-Selinene	-	-	-	-	-	-	0.1	0.1	0.2	0.1
1496	1497	*epi*-Cubebol	-	-	tr	0.1	0.1	-	-	-	-	-
1498	1497	α-Muurolene	0.1	0.1	tr	0.1	0.1	-	0.1	0.1	0.1	-
1504	1504	(*E*,*E*)-α-Farnesene	-	-	-	-	-	0.1	0.1	tr	tr	0.1
1512	1512	γ-Cadinene	0.2	0.2	0.1	0.2	0.3	-	0.1	0.2	0.4	tr
1517	1518	δ-Cadinene	0.4	0.4	0.2	0.3	0.4	tr	0.3	0.5	0.6	0.1
1532	1536	*trans*-Cadina-1,4-diene	-	-	-	-	-	-	-	-	0.1	-
1537	1540	Selina-4(15),7(11)-diene	-	-	-	-	-	-	0.1	0.1	0.1	0.1
1540	1541	α-Calacorene	-	-	-	-	0.1	-	-	-	-	-
1542	1542	Selina-3,7(11)-diene	-	-	-	-	-	-	0.2	0.1	0.1	0.2
1545	1548	Elemicin	-	-	-	0.1	0.1	-	-	-	-	-
1559	1557	Germacrene B	-	-	-	-	-	-	0.1	0.1	0.1	0.1
1560	1560	Dodecanoic acid	-	-	0.1	0.2	0.2	-	-	-	-	-
1562	1562	(*E*)-Nerolidol	-	-	-	-	-	-	0.1	-	-	-
1577	1578	Spathulenol	0.2	0.1	-	-	0.1	-	-	-	0.1	-
1582	1587	Caryophyllene oxide	-	-	-	-	tr	-	tr	-	tr	0.1
1586	1582	*epi*-Globulol	0.3	0.2	-	-	0.2	-	-	-	0.3	-
1592	1593	(7*Z*)-Tetradecenal	-	-	-	0.1	-	-	-	-	-	-
1593	1593	Ethyl laurate	-	-	0.1	-	-	-	-	-	-	-

RI_{calc}	RI_{db}	Compounds	A1	A2	A3	A4	A5	B1	B2	B3	B4	B5
1596	1593	Guaiol	-	-	-	-	-	0.4	0.2	0.1	0.2	0.4
1613	1612	5-*epi*-7-*epi*-β-Eudesmol	-	-	-	-	-	-	-	-	-	0.1
1615	1616	1,10-di-*epi*-Cubenol	-	-	-	-	-	-	-	-	0.1	-
1627	1628	1-*epi*-Cubenol	-	-	tr	0.1	0.1	-	0.1	tr	0.2	-
1628	1627	Labdanol	-	-	tr	0.1	0.1	-	-	-	-	-
1631	1632	γ-Eudesmol	-	-	-	-	-	0.3	0.1	0.1	0.2	0.5
1633	1634	*cis*-Cadin-4-en-7-ol	-	-	tr	-	-	-	0.1	0.1	-	0.2
1642	1643	τ-Cadinol	0.2	0.1	tr	0.1	0.2	-	0.1	0.3	0.5	-
1644	1644	τ-Muurolol	0.2	0.1	0.1	0.1	0.1	-	0.1	0.1	0.2	-
1647	1651	α-Muurolol (= δ-Cadinol)	0.1	0.1	tr	0.1	0.1	-	0.1	0.1	0.3	-
1655	1655	α-Eudesmol	-	-	-	-	-	1.1	0.8	0.3	-	2.0
1656	1655	α-Cadinol	0.4	0.3	0.1	0.3	0.4	-	-	0.7	1.2	-
1665	1664	Bulnesol	-	-	-	-	-	0.2	0.1	0.1	0.1	0.2
1671	1672	(10*E*)-Tetradecenol	-	-	tr	-	tr	-	-	-	-	-
1696	1697	2-Pentadecanone	-	-	tr	-	tr	-	-	-	-	-
1698	1698	Juniper camphor	-	-	-	-	-	-	0.1	0.1	0.1	0.1
1724	1723	Methyl myristate	-	-	0.2	-	0.1	-	-	-	-	-
1746	1746	Isoamyl (*E*)-cinnamate	-	-	-	-	0.1	-	-	-	-	-
1757	1758	Myristic acid	tr	0.1	0.3	0.2	0.2	-	-	-	-	-
1764	1769	Benzyl benzoate	-	-	0.2	-	0.1	-	-	-	-	0.1
1769	1763	(*Z*)-Pentadecenol*	-	-	0.1	-	tr	-	-	-	-	-
1791	1791	Ethyl myristate	0.1	0.1	0.9	0.2	0.4	tr	tr	tr	0.1	0.1
1816	1817	Hexadecanal	-	-	0.1	0.1	0.1	-	-	-	-	-
1852	1856	Phenethyl benzoate	-	-	0.2	-	tr	-	-	-	-	-
1865	1869	Benzyl salicylate	0.1	0.1	0.1	0.1	0.1	-	-	-	-	-
1878	1878	(2*E*)-Hexadecenal	-	-	-	tr	0.1	-	-	-	-	-
1892	1983	Ethyl pentadecanoate	-	-	0.1	-	0.1	-	-	-	-	-
1896	1894	Methyl 9,12-hexadecadienoate	-	-	0.1	-	0.1	-	-	-	-	-
1900	1898	Methyl palmitoleate	0.1	0.1	0.2	0.1	0.1	-	-	-	-	-
1905	1913	Methyl (11*Z*)-hexadecenoate	-	-	0.1	-	tr	-	-	-	-	-
1924	1925	Methyl palmitate	-	-	0.2	0.1	0.1	-	-	-	-	-
1963	---	Ethyl 9,12-hexadecadienoate	-	-	0.7	0.2	0.3	-	-	-	-	-
1968	1971	Ethyl palmitoleate	0.4	0.3	2.5	0.7	1.2	tr	-	-	-	-
1973	---	Ethyl (11Z)-hexadecenoate	0.1	0.1	0.8	0.1	0.2	-	-	-	-	-

RI_{calc}	RI_{db}	Compounds	A1	A2	A3	A4	A5	B1	B2	B3	B4	B5
1981	1983	(*E*,*Z*)-Geranyl linalool	-	-	-	0.1	0.1	-	-	-	-	-
1990	1993	Ethyl palmitate	0.2	0.1	2.7	0.6	1.2	0.2	0.1	0.1	0.5	0.3
2076	2075	(10*Z*)-Heptadecenoic acid	-	-	-	-	0.2	-	-	-	-	-
2143	2135	Isobutyl palmitate	-	-	-	-	0.2	-	-	-	-	-
2163	2159	Linolenic acid	-	-	-	-	0.2	-	-	-	-	-
2179	2182	Phenethyl cinnamate	-	-	-	0.1	-	-	-	-	-	-
2300	2300	Tricosane	tr	tr	tr	0.1	0.2	tr	-	tr	-	-
2500	2500	Pentacosane	0.1	0.1	0.4	0.4	0.4	tr	tr	tr	tr	tr
2700	2700	Heptacosane	0.3	0.4	0.9	1.7	1.1	0.1	tr	0.1	0.1	0.2
		Monoterpene hydrocarbons	38.4	39.3	45.0	41.8	36.3	56.3	68.3	47.0	64.7	49.6
		Oxygenated monoterpenoids	53.3	51.4	24.4	35.2	45.8	27.1	19.5	43.7	20.5	32.0
		Sesquiterpene hydrocarbons	1.4	1.2	0.4	1.2	1.6	0.3	2.0	2.1	2.7	1.4
		Oxygenated sesquiterpenoids	1.3	0.9	0.2	0.5	1.2	2.1	1.8	2.0	3.5	3.7
		Diterpenoids	0.0	0.0	0.0	0.1	0.1	0.0	0.0	0.0	0.0	0.0
		Benzenoid aromatics	0.7	1.4	14.2	6.9	2.7	10.7	3.7	2.3	3.5	7.4
		Others	4.2	5.0	15.2	13.1	11.3	3.1	4.1	2.5	4.4	5.4
		Total identified	99.4	99.2	99.4	98.8	98.9	99.6	99.4	99.6	99.4	99.5
		Monoterpenoids										
		Acyclic	17.3	19.9	16.2	17.8	14.6	12.0	16.3	9.8	15.7	13.0
		Camphane	0.1	0.1	0.1	0.1	0.1	0.6	0.5	0.2	0.5	0.3
		Menthane	67.1	64.6	45.1	50.9	61.9	45.8	44.3	65.9	44.2	52.6
		Pinane	4.0	3.2	4.6	4.4	3.4	19.7	20.5	7.8	18.5	10.8
		Thujane	2.2	1.8	2.6	2.1	1.1	4.2	4.3	5.5	4.7	3.5
		Sesquiterpenoids										
		Acyclic	0.0	0.0	0.0	0.0	0.0	0.2	0.3	0.1	0.2	0.1
		Aromadendrane	0.7	0.6	0.0	0.1	0.5	0.7	0.3	0.5	0.8	1.0
		Cadinane	2.0	1.5	0.6	1.4	2.1	0.0	1.1	2.5	4.0	0.4
		Caryophyllane	0.0	0.0	0.0	0.0	0.0	0.0	0.2	0.0	0.1	0.1
		Elemane/Germacrane	0.0	0.0	0.0	0.0	0.0	0.0	0.3	0.2	0.2	0.2
		Eudesmane	0.0	0.0	0.0	0.0	0.0	1.5	1.6	0.9	0.9	3.3
		Fatty acid derivatives	2.1	2.2	11.9	6.5	7.4	1.2	3.3	1.9	3.5	3.1

RI_{calc} = Retention index determined with respect to a homologous series of *n*-alkanes. RI_{db} = Reference retention index values from the databases. tr = trace (< 0.05%).

Supplementary Table S2. Pearson correlation between major essential oil components and polyphenolic components in *Eriodictyon californicum* (California yerba santa).

	**1,8-Cineole**	**(*Z*)-β-Ocimene**	**Terpinen-4-ol**	***α*-Pinene**	**β-Phellandrene**	***γ*-Terpinene**	**Ethyl (*E*)-cinnamate**	***α*-Terpineol**	***p*-Cymene**	**β-Pinene**	***α*-Terpinene**	**(*E*)-β-Ocimene**	**Limonene**	**(2*E*)-Hexenal**	**Myrcene**	***cis*-*p*-Menth-2-en-1-ol**	***α*-Thujene**	**Isobutyric acid**	**Terpinolene**
Rosmarinic acid	0.195	-0.053	0.124	-0.005	-0.380	0.004	0.452	0.129	0.194	-0.001	0.065	-0.412	-0.271	-0.493	-0.502	-0.006	-0.030	-0.176	-0.038
Salvianolic acid H	-0.120	0.456	**0.675**	-0.557	**-0.666**	**0.645**	0.397	-0.006	**0.702**	-0.563	0.468	-0.405	**-0.688**	-0.537	-0.589	0.616	-0.422	0.583	0.518
Melitric acid A	-0.388	-0.356	0.166	0.433	0.399	0.273	-0.055	-0.428	-0.129	0.479	0.447	0.292	0.502	0.526	0.366	0.079	**0.761**	-0.428	0.405
Eriodictyol	0.132	0.473	0.267	**-0.632**	-0.608	0.351	-0.224	0.297	0.281	-0.605	0.281	-0.318	-0.536	-0.374	-0.173	0.058	-0.305	**0.817**	0.351
Luteolin	-0.228	-0.480	0.117	0.431	0.527	0.326	-0.032	-0.368	-0.343	0.469	0.490	0.220	0.551	0.477	0.228	0.103	**0.866**	**-0.764**	0.388
6-Methoxynaringenin	0.147	0.047	0.035	-0.013	0.076	0.318	-0.030	-0.045	-0.317	0.020	0.391	-0.054	0.009	0.088	-0.209	0.026	0.435	-0.499	0.252
Naringenin	0.362	0.604	0.227	**-0.802**	**-0.690**	0.138	-0.061	0.452	0.402	**-0.795**	-0.060	-0.542	**-0.875**	**-0.681**	**-0.726**	0.200	**-0.756**	**0.657**	-0.021
Hispidulin	0.254	0.620	0.155	**-0.691**	**-0.680**	0.061	-0.131	0.429	0.374	**-0.688**	-0.098	-0.325	**-0.705**	-0.576	-0.360	0.049	**-0.727**	**0.884**	-0.004
Homoeriodictyol	-0.017	0.020	0.394	-0.457	-0.367	0.342	-0.135	0.174	0.369	-0.440	0.286	-0.372	-0.308	-0.319	-0.132	0.221	-0.149	0.607	0.370
Jaceosidin	0.159	0.415	0.002	-0.491	-0.567	-0.003	-0.148	0.337	0.236	-0.502	-0.070	-0.219	-0.413	-0.348	0.061	-0.164	-0.470	**0.922**	0.029
Hesperetin	0.396	0.042	-0.028	-0.291	-0.030	0.243	-0.250	0.230	-0.359	-0.253	0.278	-0.316	-0.177	-0.111	-0.377	-0.053	0.260	-0.351	0.146
Eriolic acid C	0.552	**0.784**	-0.022	**-0.854**	**-0.817**	0.057	-0.336	**0.691**	0.132	**-0.833**	-0.104	-0.446	**-0.882**	**-0.720**	-0.518	-0.171	**-0.802**	**0.860**	-0.043
Sterubin	-0.451	**-0.805**	0.054	**0.746**	**0.732**	0.016	0.211	-0.575	-0.181	**0.747**	0.214	0.322	**0.832**	**0.634**	0.475	0.107	**0.890**	**-0.813**	0.138
Sakuranetin	-0.109	-0.425	0.081	0.266	0.241	0.195	0.359	-0.300	-0.062	0.246	0.265	-0.159	0.221	0.181	-0.107	0.174	0.470	-0.606	0.147
Pinocembrin	0.208	0.379	0.226	**-0.676**	-0.592	0.055	-0.091	0.399	0.488	**-0.681**	-0.143	-0.486	**-0.712**	-0.601	-0.499	0.185	**-0.773**	**0.784**	-0.034

Supplementary Table S3. Instrument details for the gas chromatographic analyses of *Eriodictyon californicum* (California yerba santa) essential oils.

**Gas Chromatography - Mass Spectrometry (GC-MS)**	
Instrument	Shimadzu GC-MS-QP2010 Ultra (Shimadzu Scientific Instruments, Columbia, MD, USA)
GC Column	Zebron ZB-5ms fused silica capillary column (60 m × 0.25 mm × 0.25 μm film thickness) (Phenomenex, Torrance, CA, USA)
MS Detector Conditions	Electron impact (EI) mode, electron energy = 70 eV, a scan = 40–400 atomic mass units, scan rate = 3.0 scans/second
Carrier Gas, Conditions	Helium, column head pressure = 208.3 kPa, flow rate = 2.00 mL/min
Injector, Detector Temperatures	Injector temperature = 260 °C, interface temperature = 260 °C, ion source temperature = 260 °C
GC Oven Temperature Program	Initial temperature = 50 °C, ramp 2 °C/min to 260 °C, hold 260 °C for 5 min
Sample Concentration, Volume Injected	5% (in dichloromethane), 0.1 μL volume
Split Mode	24.5 : 1.0
**Chiral Gas Chromatography - Mass Spectrometry**	
Instrument	Shimadzu GCMS-QP2010S (Shimadzu Scientific Instruments, Columbia, MD, USA)
GC Column	Restek B-Dex 325 chiral GC column (30 m × 0.25 mm × 0.25 μm film thickness) (Restek Corp., Bellefonte, PA, USA)
MS Detector Conditions	Electron impact (EI) mode, electron energy = 70 eV, a scan = 40–400 atomic mass units, scan rate = 3.0 scans/second
Carrier Gas, Conditions	Helium, column head pressure = 53.6 kPa, flow rate = 1.00 mL/min
Injector, Detector Temperatures	Injector temperature = 240 °C, interface temperature = 240 °C, ion source temperature = 240 °C
GC Oven Temperature Program	Initial temperature = 50 °C, hold for 5 min, ramp 1 °C/min to 100 °C, ramp 2 °C/min to 220 °C
Sample Concentration, Volume Injected	5% (in dichloromethane), 0.3 μL volume
Split Mode	24.0 : 1.0

Supplementary Table S4. UHPLC/DAD/Q-ToF quantification of targeted compounds in *Eriodictyon californicum* samples.

No.	Compound	Formula	DAD (nm)	Accurate Mass	Quantification Method	$[M-H]^-$
1	Rosmarinic acid	$C_{18}H_{16}O_8$	330	360.0845	ESI (-)	359.0772
2	Salvianolic acid H	$C_{27}H_{22}O_{12}$	330	538.1111	ESI (-)	537.1038
3	Melitric acid A	$C_{27}H_{22}O_{12}$	330	538.1111	ESI (-)	537.1038
4	Eriodictyol	$C_{15}H_{12}O_6$	288	288.0634	ESI (-)	287.0561
5	Luteolin	$C_{15}H_{10}O_6$	350	285.0405	ESI (-)	285.0421
6	Naringenin	$C_{15}H_{12}O_5$	288	272.0685	ESI (-)	271.0612
7	Hispidulin	$C_{16}H_{12}O_6$	330	300.0634	ESI (-)	299.0561
8	Homoeriodictyol	$C_{16}H_{14}O_6$	288	302.0790	ESI (-)	301.0718
9	Jaceosidin	$C_{17}H_{14}O_7$	340	330.0740	ESI (-)	329.0667
10	6-Methoxynaringenin	$C_{16}H_{14}O_6$	288	302.0790	ESI (-)	301.0718
11	Hesperetin	$C_{16}H_{14}O_6$	288	302.0790	ESI (-)	301.0718
12	Eriolic acid C	$C_{22}H_{30}O_5$	260	374.2093	ESI (-)	373.2020
13	Sterubin	$C_{16}H_{14}O_6$	288	302.0790	ESI (-)	301.0718
14	Sakuranetin	$C_{16}H_{14}O_5$	288	286.0841	ESI (-)	285.0768
15	Pinocembrin	$C_{15}H_{12}O_4$	288	256.0736	ESI (-)	255.0663

All compounds were identified using standard compounds.

Rosmarinic Acid

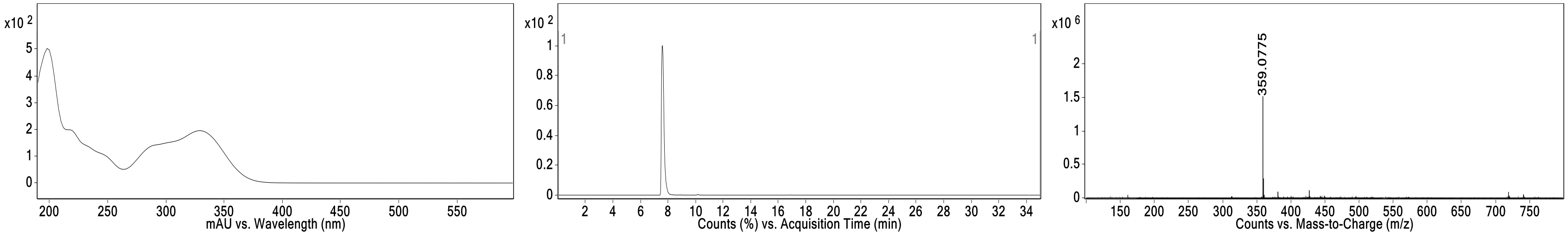

Salvianolic Acid H

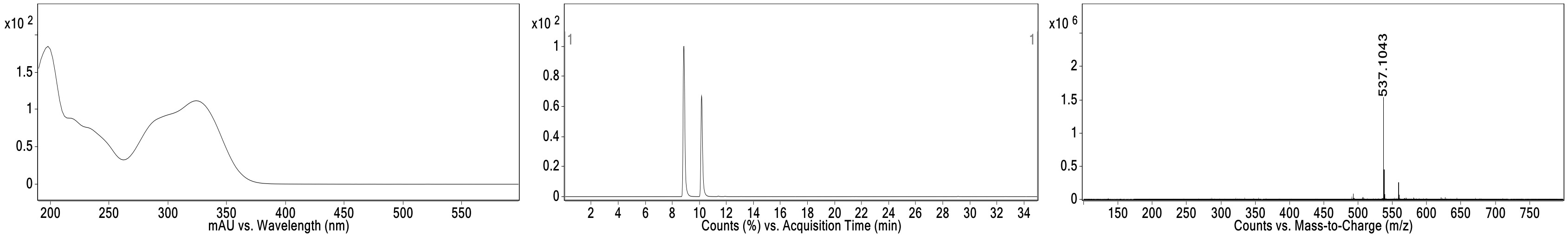

Melitric Acid A

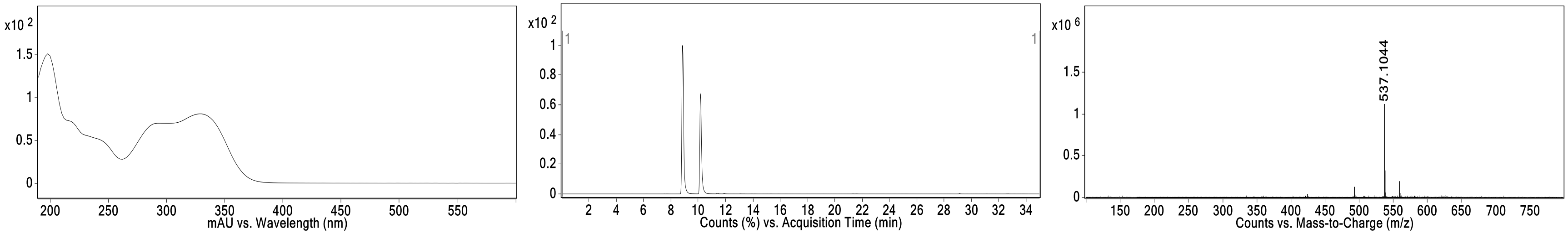

Eriodictyol

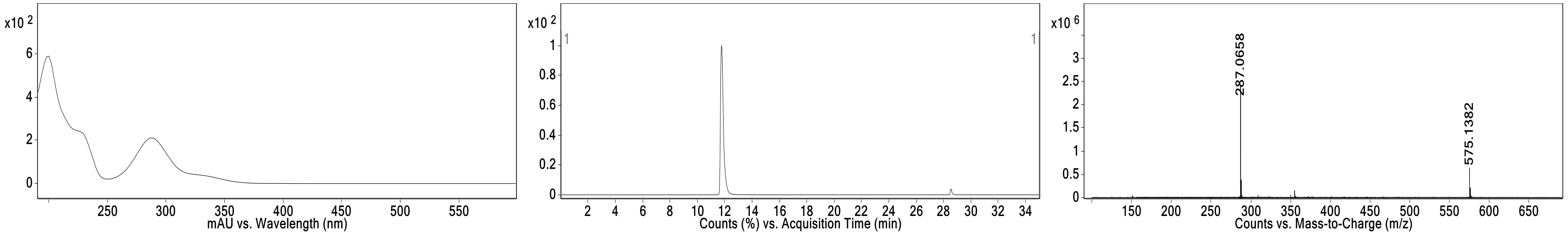

Luteolin

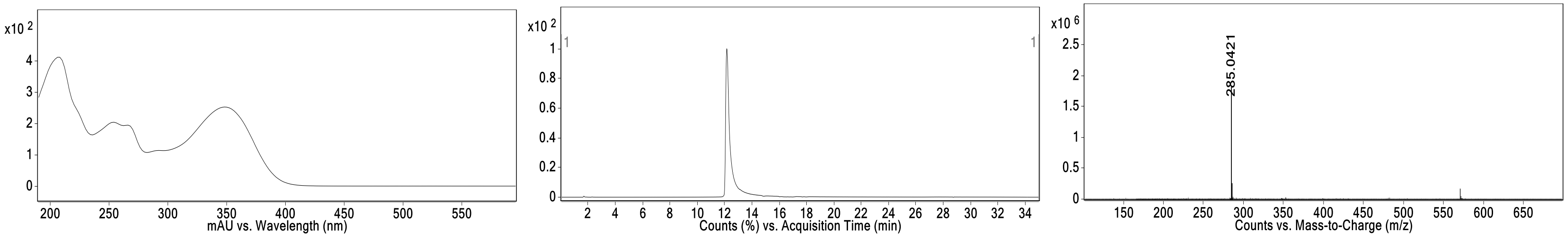

Naringenin

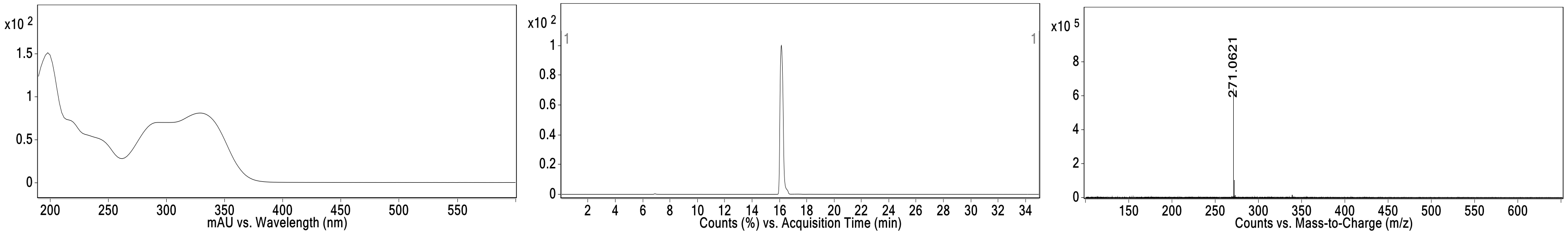

Hispidulin

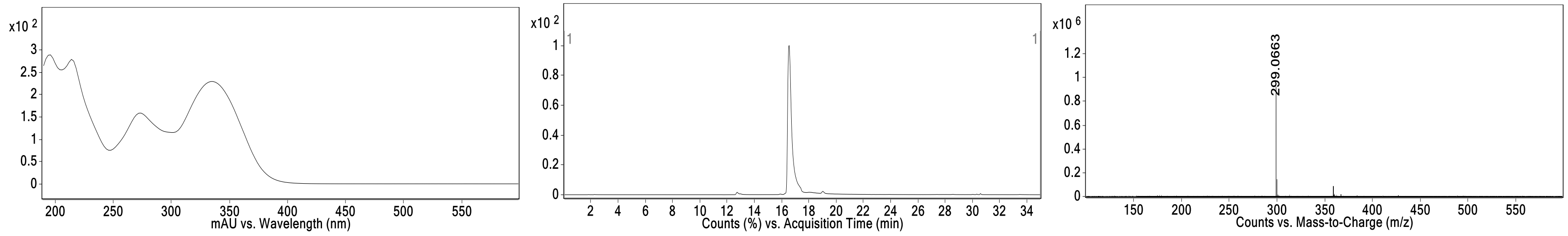

Homoeriodictyol

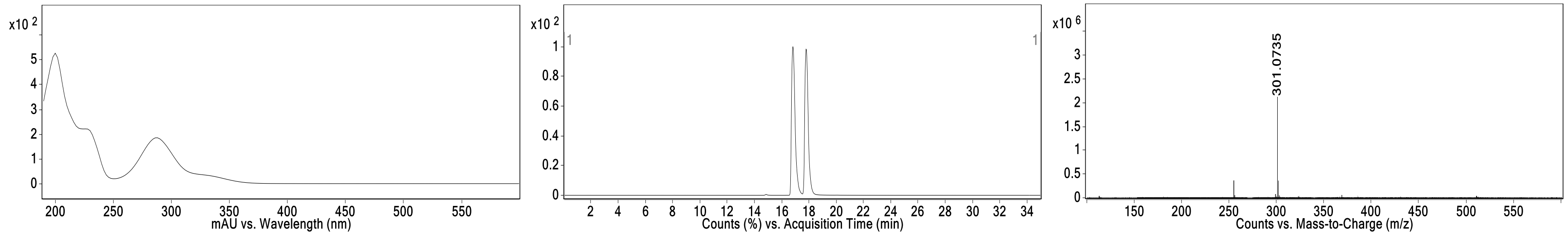

Jaceosidin

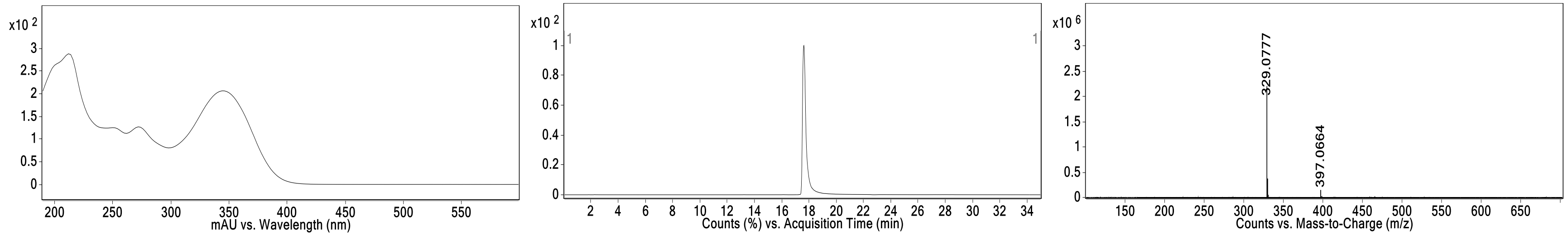

6-Methoxynaringenin

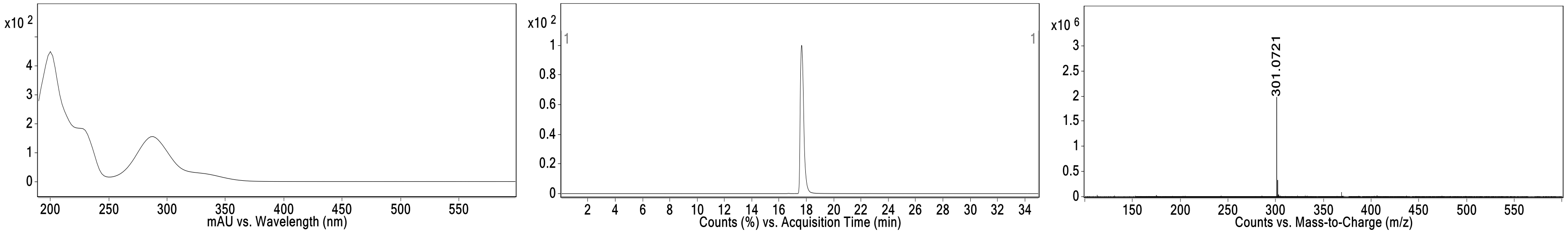

Hesperetin

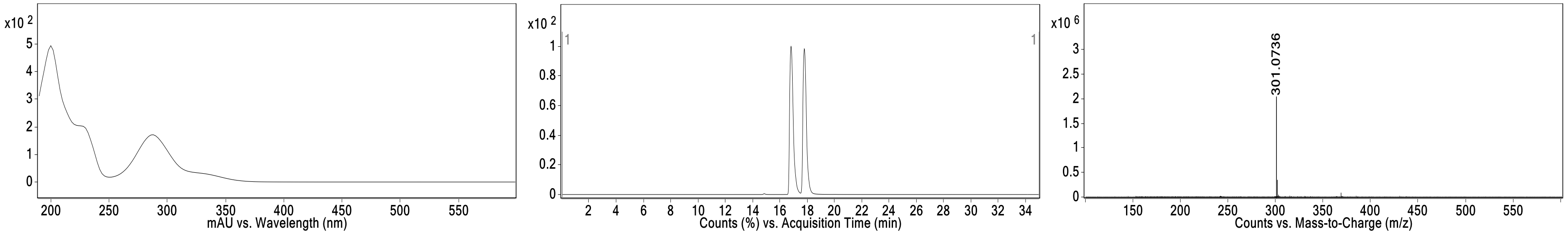

Eriolic Acid C

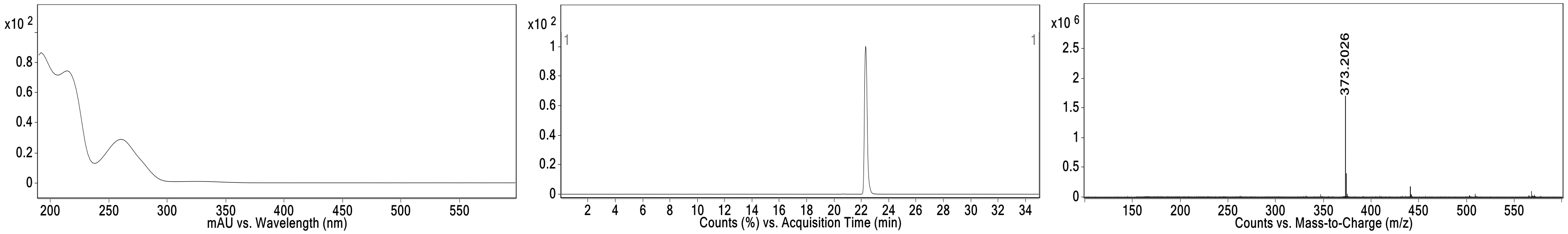

Sterubin

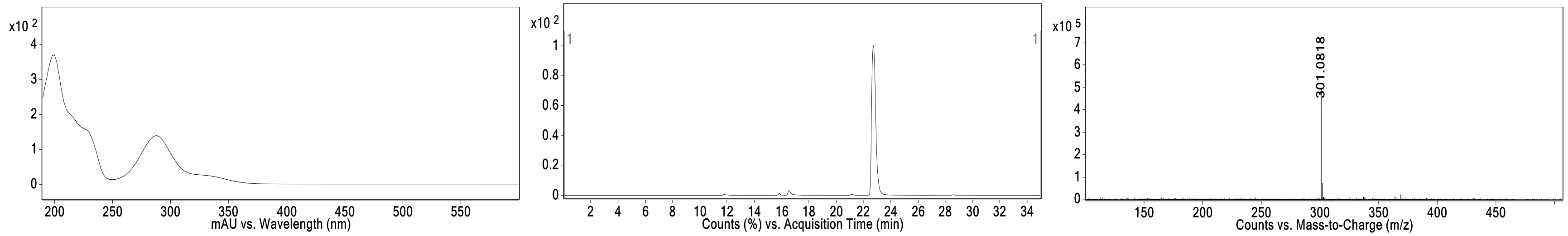

Sakuranetin

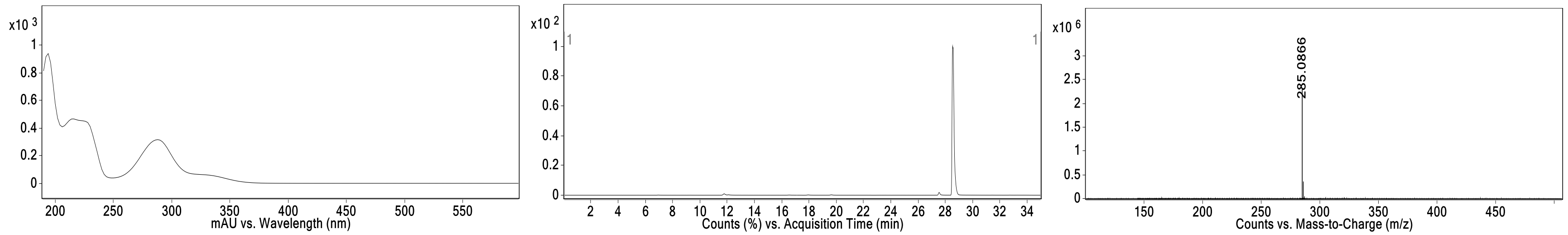

Pinocembrin (

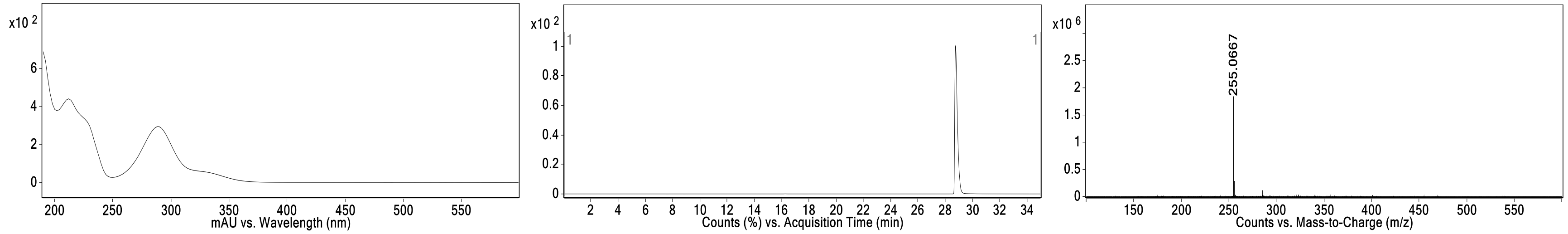

Supplementary Figure S1. UV spectra, extracted ion chromatograms (EICs) in ESI^{-} mode, and ToF MS spectra for each compound quantified in *Eriodictyon californicum*.
